# The Curette

**Published:** 1909-10

**Authors:** 


THE CURETTE.—With many physicians the first thought in uterine bleeding is the curette. The hemorrhage is frequently due to lack of tonicity of the blood-vessels and muscular tissues of the uterine walls and to curette in these cases is unnecessary and frequently dangerous. The value of viburnum as presented by Hayden's Viburnum Compound in these cases has been proven conclusively by years of clinical experience. It imparts tone to the relaxed uterine blood-vessels and walls and in many cases makes curettement with its attending dangers of infection and perforation unnecessary.

